# Immobilized Osteopontin Enhances Adhesion but Suppresses Cytokine Release of Anti-IgE Activated Human Mast Cells

**DOI:** 10.3389/fimmu.2018.01109

**Published:** 2018-05-22

**Authors:** Chun Wai Ng, Issan Yee San Tam, Sze Wing Sam, Yangyang Yu, Hang Yung Alaster Lau

**Affiliations:** ^1^Faculty of Medicine, School of Biomedical Sciences, The Chinese University of Hong Kong, Hong Kong, Hong Kong; ^2^Department of Physiology, Shenzhen University Health Science Center, Shenzhen, China

**Keywords:** osteopontin, mast cell, cytokines, IgE, adhesion

## Abstract

Osteopontin (OPN) is an Arg-Gly-Asp (RGD)-containing extracellular matrix protein which is upregulated in inflamed tissues and has been reported to modulate mast cell activities in mice. Due to the known heterogeneity among mast cells of different species and the important roles of mast cells in allergic reactions, we investigated the effects of human OPN (hOPN) on human mast cell activities. Mature primary human cultured mast cells (HCMC) were derived from peripheral blood CD34^+^ progenitors and the modulation of their activation by soluble and plate-bound immobilized hOPN were examined by studying their release of inflammatory mediators (histamine, IL-5, IL-8, TNF-α, and VEGF) and matrix adhesion following stimulation by anti-IgE. Immobilized hOPN enhanced the adhesion, but suppressed the release of IL-5, IL-8, and TNF-α of anti-IgE-activated HCMC while soluble hOPN failed to demonstrate any significant effects. By employing cyclic RGD peptide and neutralizing antibodies against different classes of integrin and CD44, we demonstrated that the interaction of immobilized hOPN and HCMC was mediated by the RGD domain of hOPN and integrin but not CD44 on HCMC. Our results suggest that immobilized hOPN anchored to extracellular matrix can regulate adaptive immunity in humans by retaining mast cells at the site of inflammation and suppressing anti-IgE-induced cytokine release from HCMC.

## Introduction

Mast cells are immune effectors abundantly located in tissues that interface with external environment such as the respiratory tract where they are found around peripheral nerves, blood vessels, and lymph vessels. They are essential in the pathogenesis of allergic reactions through the dimerization of cell surface high affinity IgE receptors (Fc_ε_RI) mediated by crosslinking of receptor bound IgE molecules by allergens (1, 2). Upon activation of Fc_ε_RI, mast cells immediately discharge the contents of their cytoplasmic granules, such as histamine, heparin, and neutral proteases to the immediate environment through the process of degranulation which is then followed by the *de novo* synthesis of pro-inflammatory lipid mediators and cytokines, leading to acute and delayed inflammatory responses. Apart from mediating the signs and symptoms of allergic reactions, these mediators also contribute to a wide range of pathophysiological processes, such as wound repairing, tissue remodeling, and tumor progression. The spectrum of mediators expressed in mast cells and how they are released determine the functions of mast cells and are greatly influenced and regulated by the myriad of factors locally produced at inflammatory sites. The extracellular matrix protein, osteopontin (OPN) has recently been proposed to modulate mast cell functions (3).

Osteopontin is a negatively charged acid-rich, phosphorylated glycoprotein that is produced in a variety of tissues and appears in different body fluids. It can function as both an immobilized matricellular protein and a soluble cytokine and is thus involved in a wide spectrum of physiological as well as pathological processes in multiple organs and tissues, including biomineralization, inflammation, leukocyte recruitment, and cell survival (4). Structurally, human OPN (hOPN) has two integrin binding motifs: a typical arginine–glycine–aspartate (RGD) integrin binding domain common to matrix protein and a cryptic serine–valine–valine–tyrosine–glycine–leucine–arginine motif that binds to integrin only after cleavage of native OPN by thrombin. In addition to interacting with integrin αv(β1, β2, or β5) and (α4, α5, α8, or α9)β1, OPN can also interact with the non-integrin cell surface receptor, CD44 (5).

Osteopontin was independently identified in activated CD4^+^ T cells as the cytokine Eta-1 (early T cell activation-1) and has since been recognized as an important regulator of immune cell functions (6). OPN promotes acute proinflammatory responses upon tissue injury, such as monocyte recruitment, macrophage differentiation, and phagocytosis (7). It has been shown to be chemoattractant to a variety of immune cells and was able to induce migrations of T cells and macrophages. It was demonstrated that migration induced by OPN was mediated through CD44 and αvβ3. In addition to acute inflammation, synthesis of OPN by activated T cells is an essential early step in the development of type-1 immunity which is the hallmark of chronic inflammatory conditions. In the macrophage, OPN enhances the production of Th1 cytokines such as IL-12 through integrin engagement and inhibits the expression of IL-10 through CD44 engagement. OPN also modulates chronic inflammatory responses through the retention of macrophages at sites of injury and pathogen infection to limit the propagation of the infection (8). OPN is believed to play an important role in orchestrating local immune responses against environmental insults by providing acute first-line anti-infection defense, while limiting inflammation and injury induced tissue damage and promoting wound healing (9).

Similar to mast cells, OPN expression is increased in response to tissue injury, infection, and in many diseases characterized by chronic inflammation, including asthma, Crohn disease, and rheumatoid arthritis (9, 10). While mast cells and OPN have separately been implicated in the development of the same pathophysiological conditions, their interaction has not been investigated until recently. Nagasaka et al. demonstrated in the rodents that OPN induced chemotactic migration of mast cells and enhanced IgE-mediated degranulation of mast cells through binding to CD44 and the αv integrin (3). Furthermore, the authors demonstrated that OPN regulated the magnitude of IgE-mediated passive cutaneous anaphylaxis reaction through modulation of skin mast cell degranulation. Despite of employing animal models, this report has clearly identified OPN as a new player in mast cell biology and directly illustrated that the modulation of mast cell functions by OPN contributes significantly to inflammation and related conditions. However, mouse mast cells are known to be heterogeneous to human mast cells in many aspects. It is hence essential to evaluate and further investigate such interaction in human mast cells for clinically relevant results. The current study aimed to define the modulation of human mast cell activities by OPN with the focus of anti-IgE-induced mast cell activation.

## Materials and Methods

### Human CD34^+^ Stem Cell-Derived Mast Cells

Fresh buffy coats were obtained from healthy blood donors, as anonymously provided by the Hong Kong Red Cross. Ethical approvals for all blood sources and processes used in this study were approved by the Joint Chinese University of Hong Kong—New Territories East Cluster Clinical Research Ethics Committee (The Joint CUHK-NTEC CREC). All experiments were carried out in accordance with the approved guidelines and regulations. Mature primary human cultured mast cells (HCMC) were derived from progenitors isolated from fresh buffy coat collected from the Hong Kong Red Cross as previously described (11). Briefly, isolated progenitors were first cultured with stem cell factor (SCF) and IL-6 in serum-free methylcellulose (StemCell Technologies, Vancouver, BC, Canada) for 6 weeks with IL-3 added in the first 2 weeks only. This was followed by culturing for 6 weeks in Iscove’s modified Dulbecco’s medium with 10% fetal bovine serum (FBS) and 1% penicillin/streptomycin (GIBCO, Carlsbad, CA, USA) supplemented with SCF and IL-6. HCMC were identified with May Grunwald–Giemsa (Merck, Darmstadt, Germany) staining of cytoplasmic granules as well as immunocytochemical staining of human mast cell tryptase using a monoclonal anti-human tryptase antibody (Chemicon, Billerica, MA, USA) and the alkaline APAAP kit from Dako (Carpinteria, CA, USA). At the end of the 12 weeks culturing period, all the cells harvested were confirmed to be mature HCMC by staining positively for human mast cell tryptase. The cells were sensitized with 0.5 µg/ml myeloma IgE (Merck, Darmstadt, Germany) overnight before experiment. IL-3, IL-6, and SCF were purchased from Peprotech Asia, Rehovot, Israel.

### Cell Adhesion

Cell adhesion assay was performed as described by Ariel et al. (12). Briefly, 96-well Nunc Maxisorp flat bottom microtiter plates (NUNC, Naperville, IL, USA) were coated with hOPN (Lee Biosolutions, Maryland Heights, MO, USA) by adding 50 µl of hOPN at various concentrations in phosphate-buffered saline (PBS) to the wells and were incubated for 2 h at 37°C. The wells were then extensively rinsed by washing three times with PBS and then incubated with 3% bovine serum albumin (BSA; Sigma, St. Louis, MO, USA) in PBS for 1 h at 37°C to block nonspecific protein binding sites. All experiments included control wells coated only with BSA by incubation with only 3% BSA for 1 h at 37°C. IgE-sensitized HCMC (5 × 10^4^) suspended in 100 µl IMDM with 100 ng/ml SCF and 10% FBS were then seeded into both control and hOPN-coated culture wells. Following incubation in a CO_2_ incubator for 1 h, the cells were further incubated with 1 µg/ml of anti-IgE (Sigma, St. Louis, MO, USA) for 2 h. Finally, the wells were rinsed three times with PBS to remove non-adherent cells. The density of adhered cells was determined fluorometrically following staining of cellular nucleic acids with the CyQUANT Cell Proliferation Assay kit (Invitrogen, Carlsbad, CA, USA). Results are expressed as the percentage of total cells adhered to culture surface which was calculated with reference to the fluorescence emitted by 5 × 10^4^ HCMC.

### HCMC Stimulation and Measurement of Secreted Mediators and Cytokines

IgE-sensitized HCMC were suspended in IMDM with 100 ng/ml SCF and 10% FBS at a cell density of 5 × 10^5^ cells/ml. The cells were then incubated with hOPN either in suspension or immobilized on culture plate surface for 1 h before being activated by anti-IgE. For histamine release, HCMC were incubated with anti-IgE for 30 min prior to addition of ice-cold buffer followed immediately by centrifugation. The cell pellets and supernatants were collected separately and the contents of histamine were measured spectrofluorometrically using a Bran + Luebbe AutoAnalyzer 3 (Norderstedt, Germany). The percentage of total cellular histamine released into the supernatant was then calculated and expressed as [histamine release (%)] and was corrected for spontaneous release that was in general less than 5%. For the release of cytokines, HCMC were incubated with anti-IgE for 24 h and the supernatants were then collected and analyzed to determine the amounts of cytokines released by using commercially available EIA kits (BD Biosciences, Franklin Lakes, NJ, USA) and the results were presented as (total cytokine release/million cells). All incubation procedures were carried out at 37°C in 5% CO_2_ incubator.

For studying the effects of blocking antibodies or inhibitors, IgE-sensitized HCMC were pre-incubated with 1 µg/ml of the antibody or the appropriate concentration of the inhibitor for 1 h at 37°C before performing adhesion or mediator release assays. The results are expressed as the percentage of the anti-IgE induced level of mediator released that was reduced in the presence of immobilized hOPN (% inhibition). The antibodies against various potential OPN receptors are the same as those employed for immunostaining. The inhibitors include the αvβ3 integrin binding cyclic RGD peptide and the corresponding control cyclic RAD peptide (Bachem, Bubendorf, Switzerland).

### Immunostaining for the Expression of Potential OPN Receptors in HCMC

IgE-sensitized HCMC were induced to adhere to immobilized hOPN by anti-IgE as described above. The adhered cells were directly fixed with 4% formaldehyde for 15 min at room temperature. The fixed cells were washed with distilled water followed by blocking with peroxidase blocker for 5 min and then washed with PBS. The blocked cells were then incubated with 1 µg/ml of different antibodies for 1 h at room temperature followed by washing with PBS. Binding of antibody was visualized with DAKO immunostaining kit as instructed by the manufacturer. The antibodies employed for staining were those against α1, α2, α3, α4, α5, αvβ3, β1, β2, β3, β4, CD44, and CD44v6 and were clone FB12, P1E6, P1B5, P1H4, P1D6, 23C6, P4G11, P4H9, 25E11, ASC-9, G44-26, and 2F10, respectively. Antibody of α1, α2, α3, α4, α5, β1, β2, β3, and β4 were obtained from Merck Millipore, αvβ3 and CD44v6 antibody were from R&D systems and CD44 antibody was from BD Bioscience.

### Statistics

Statistical analysis was performed with Prism software (version 6.0f, Graphpad). Differences between treatment groups were analyzed with repeated measure one-way ANOVA or paired *t*-test where appropriate. Results are presented as mean ± SEM for the number of experiments reported and *p* < 0.05 was considered statistically significant. Different donors were used in each experiment.

## Results

### Immobilized but Not Soluble hOPN Regulates the Release of IL-5, IL-8, and TNF-α From Anti-IgE-Activated HCMC

**Figure 1:**
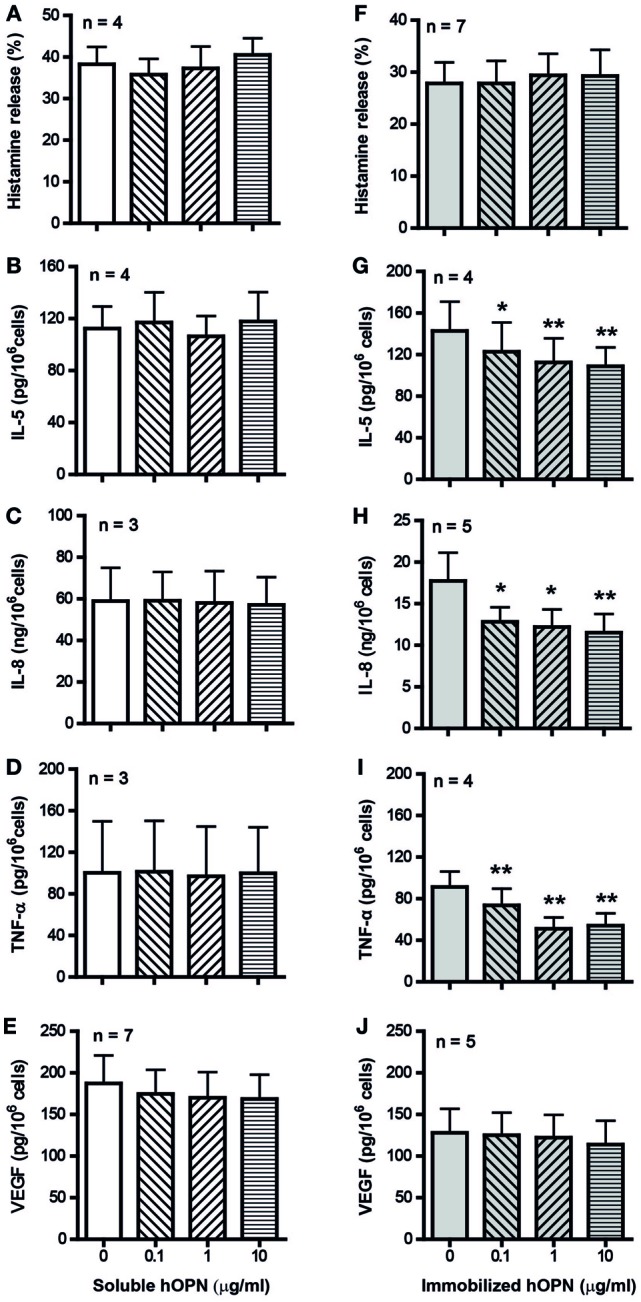
Effect of soluble and immobilized hOPN on anti-IgE-induced release of **(A,F)** histamine, **(B,G)** IL-5, **(C,H)** IL-8, **(D,I)** TNF-α, and **(E,J)** VEGF. IgE-sensitized human cultured mast cells (HCMC) were pre-incubated with different doses (0.1, 1, and 10 µg/ml) of soluble or immobilized hOPN for 1 h and then challenged by 1 µg/ml anti-IgE for 24 h. Histamine released into the supernatant and remained in cells were determined spectrofluorometrically, while the level of cytokines released into supernatants were determined by ELISA. Histamine release is expressed as the percentage of total cellular histamine released after correction for the basal spontaneous release [**(A)** 3.0 ± 1.4%, *n* = 4, **(F)** 0.9 ± 0.9%, *n* = 7], while the absolute amounts of cytokines released per million cells are reported. HCMC spontaneously released VEGF [**(E)** 136.8 ± 71.9 pg/10^6^ cells, *n* = 5, **(J)** 141.3 ± 83.9 pg/10^6^ cells, *n* = 7] but no detectable amount of IL-5, IL-8, and TNF-α. Data are presented as mean ± SEM for the number of experiments (*n*) shown. *p < 0.05, **p < 0.01 by one-way ANOVA with Dunn’s multiple comparison test when compared with release induced by anti-IgE alone.

IgE-sensitized HCMC spontaneously released around 5% of total cellular histamine and 100 ng/10^6^ cells of VEGF but no detectable levels of IL-8, TNF-α, and IL-5 in culture medium alone. While incubation with either form of hOPN did not significantly affect the basal spontaneous release of these mediators, anti-IgE activation significantly increased the release of histamine and VEGF and induced the release of IL-5, IL-8, and TNF-α. Preincubation of HCMC with soluble hOPN (1–10 µg/ml) for 1 h did not have any effect on the levels of anti-IgE-induced release of the various inflammatory mediators (Figures 1A–E). In contrast, preincubation with immobilized hOPN (1–10 µg/ml) for 1 h suppressed anti-IgE stimulated release of IL-5, IL-8, and TNF-α but had no effect on the release of histamine and VEGF (Figures 1F–J). At the maximum concentration of 10 µg/ml, immobilized hOPN reduced the levels of anti-IgE induced release of IL-5, IL-8, and TNF-α from 142.7 ± 14.1, 17,740 ± 3,402, and 91.2 ± 14.9 pg/10^6^ cells to 108.7 ± 9.1, 11,530 ± 2,225, and 54.1 ± 11.8 pg/10^6^ cells, equivalent to reduction by 35, 44, and 24%, respectively.

### Immobilized hOPN Is Able to Mediate Morphological Change and Adhesion of Anti-IgE-Activated HCMC

**Figure 2:**
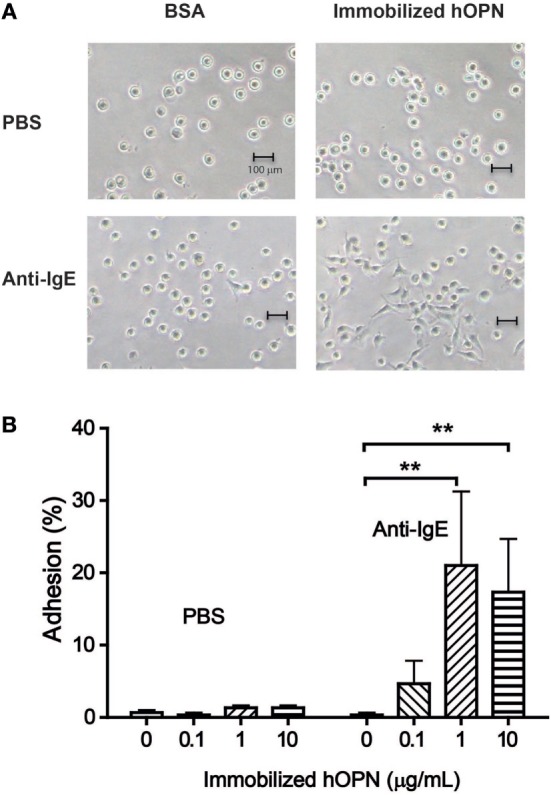
**(A)** Morphological change of human mast cells mediated by immobilized hOPN. IgE-sensitized human cultured mast cells (HCMC) were placed in culture wells pre-coated with bovine serum albumin (control) or 1 µg/ml hOPN and incubated with phosphate-buffered saline (PBS) or 1 µg/ml anti-IgE for 2 h. The images of the HCMC were taken under light microscope (200× magnification). **(B)** Adhesion of human mast cells on immobilized hOPN with or without the presence of anti-IgE. IgE-sensitized HCMC were incubated in culture wells coated with 0, 0.1, 1, and 10 µg/ml immobilized hOPN as indicated, followed by incubation with or without 1 µg/ml anti-IgE for 2 h. Following the removal of non-adherent cells by repeated rinsing, the density of cells remained in the culture well was determined by CyQUANT cell proliferation assay. Data are shown as the mean of the percentage of cells became adherent [adhesion (%)] ± SEM (*n* = 3); *p < 0.05, **p < 0.01 by one-way ANOVA with Dunn’s multiple comparison test when compared with corresponding OPN-free (PBS) value.

Following 2 h incubation, IgE-sensitized HCMC without activation appeared round in shape in both BSA-coated control wells and wells coated with hOPN coated with 1 µg/ml (Figure 2A PBS). The appearance of HCMC remained unchanged after anti-IgE activation in the BSA-coated control wells. However, significant change in HCMC morphology indicating spreading of the cells on the culture well surface coated with hOPN was observed, suggesting specific interaction between anti-IgE-activated HCMC and immobilized hOPN (Figure 2A, anti-IgE). While less than 1% of unstimulated IgE-sensitized HCMC were retained in the hOPN-coated culture wells, significant adhesion to hOPN coated culture wells was observed with anti-IgE-activated mast cells, where 4.7 ± 3.2, 21 ± 10.3, and 17.3 ± 7.4% of cells adhered to 0.1, 1, and 10 µg/ml of hOPN, respectively (Figure 2B).

### Integrin αvβ3, α5, β2, β4, and CD44 Are Expressed in HCMC and They Differentially Regulate Adhesion and Cytokine Release of Anti-IgE-Activated HCMC

**Figure 3:**
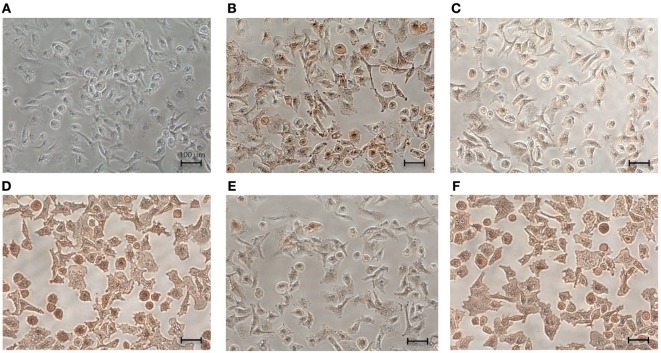
Expression of integrins and CD44 on the surface of human cultured mast cells (HCMC). IgE-sensitized HCMC were cultured on surface pre-coated with 1 µg/ml hOPN and activated by 1 µg/ml anti-IgE for 2 h. The cells were then fixed by 4% formaldehyde for 15 min at room temperature and incubated with 1 µg/ml of **(A)** control IgG antibody, **(B)** anti-integrin αvβ3 antibody, **(C)** anti-integrin α5 antibody, **(D)** anti-integrin β2 antibody, **(E)** anti-integrin β4 antibody, and **(F)** anti-CD44 antibody for 1 h at room temperature. The expressions of respective receptor were visualized by DAB immunostaining (200× magnification). The mouse monoclonal antibodies were IgG_1_ for αvβ3 and CD44, IgG_3_ for α5 and β2, and IgG_2a_ for β4. Isotype-specific controls with corresponding non-immune IgG were performed. No positive staining was observed with all the phenotype-specific controls and the typical result was presented in **(A)** for the IgG_1_ control antibody.

For elucidating the receptors on mast cells that are responsible for the interaction between HCMC and immobilized hOPN, specific monoclonal antibodies against different classes of integrin and CD44 were employed in immunostaining studies for detecting the expression of these receptors. As shown in Figure 3, monoclonal antibodies against integrin αvβ3, α5, β2, β4, and CD44 were able to stain anti-IgE-activated HCMC that adhered to immobilized hOPN, indicating that the corresponding integrins were expressed on HCMC.

**Figure 4:**
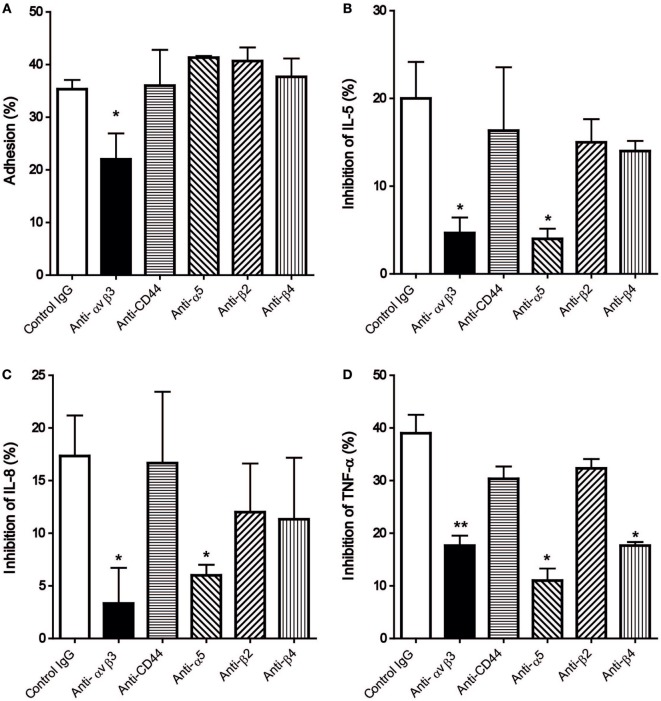
The effect of anti-integrin and anti-CD44 antibodies on **(A)** adhesion and release of **(B)** IL-5, **(C)** IL-8, and **(D)** TNF-α of human cultured mast cells (HCMC) induced by anti-IgE on immobilized hOPN. IgE-sensitized HCMC were pre-incubated with 1 µg/ml of the antibodies against integrin αvβ3, CD44, α5, β2, and β4 for 1 h at room temperature, and then added to culture wells pre-coated with 1 µg/ml of hOPN for a further 1 h incubation. The cells were then stimulated with 1 µg/ml anti-IgE for 2 or 24 h for adhesion and cytokines release, respectively. The percentage of cell adhesion was then determined as described in Figure 2. The results of cytokine release are expressed as the percentage of the anti-IgE induced level of cytokine released that was reduced in the presence of immobilized osteopontin [inhibition (%)]. Data are shown as mean ± SEM (*n* = 3); *p < 0.05, **p < 0.01 by one-way ANOVA with Dunn’s multiple comparison tests when compared with values obtained with control IgG.

As the expression of integrin αvβ3, α5, β2, β4, and CD44 was demonstrated (Figure 3), their corresponding antibodies were further employed in the subsequent studies to delineate the functional significance of these integrins. Among these antibodies, only the antibody against αvβ3 could significantly reduce the degree of HCMC adhesion to immobilized hOPN following anti-IgE activation from 35.3 ± 1.8 to 22 ± 4.9% (Figure 4A). This antibody also significantly suppressed the inhibitory efficiency of hOPN on the anti-IgE-induced release of IL-5 (20 ± 4.2 to 4.7 ± 1.8%), IL-8 (17.3 ± 3.8 to 3.3 ± 3.4%), and TNF-α (39 ± 3.5 to 17.7 ± 1.9%, *n* = 3) (Figure 4). The antibody against α5 could significantly reduce the inhibition of IL-5, IL-8, and TNF-α by immobilized hOPN to 4 ± 1.2, 6.0 ± 1.0, and 11.0 ± 2.3% respectively (Figure 4). The antibody against integrin β4 could only significantly reduce the hOPN-induced inhibition of TNF-α to 17.7 ± 0.7% (Figure 4). However, antibodies against β2 and CD44 had no effect on immobilized hOPN-mediated modulation of cytokine release from anti-IgE-activated HCMC.

**Figure 5:**
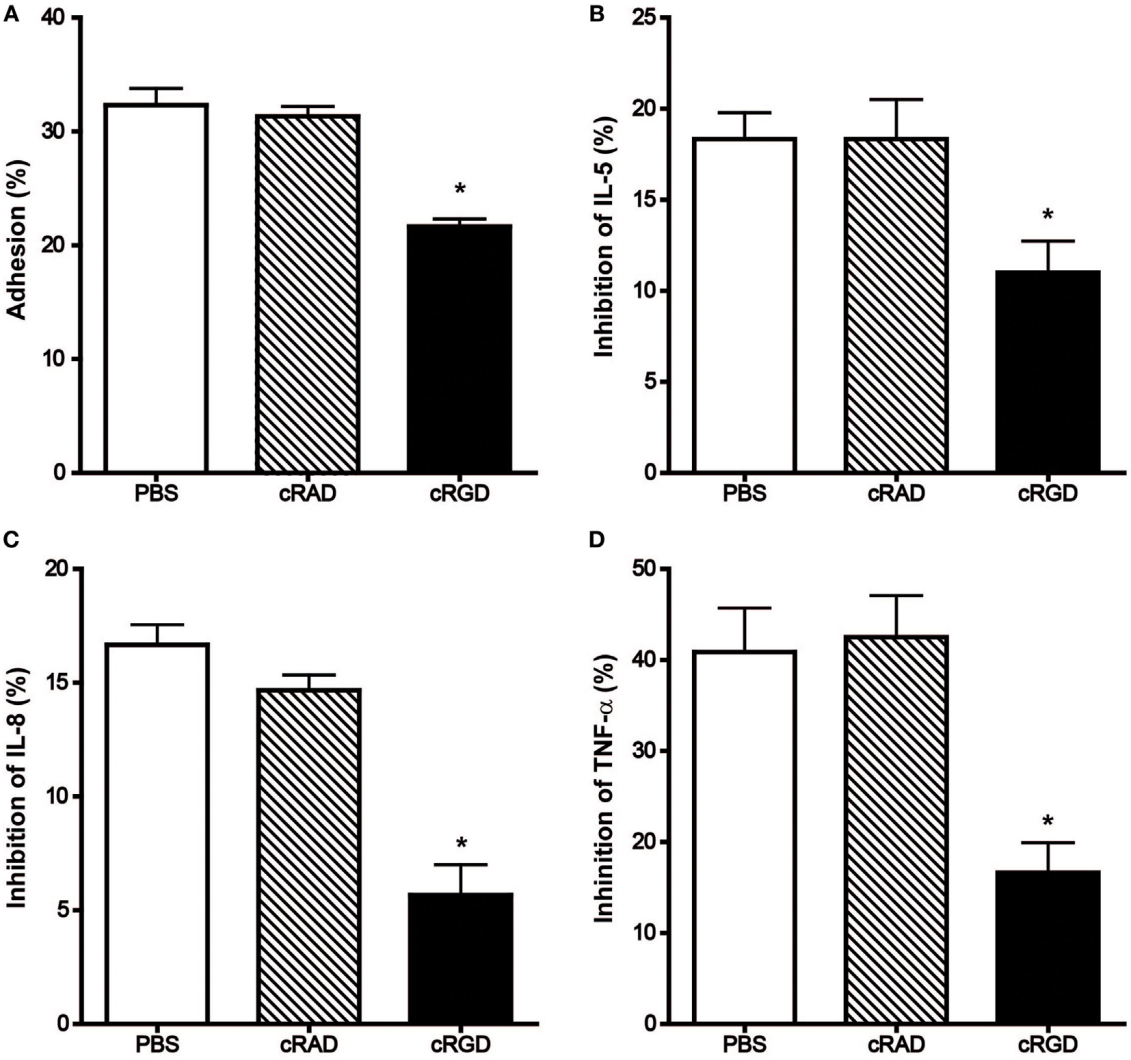
Effect of cRGD and the control peptide cRAD on the immobilized hOPN-mediated **(A)** adhesion and inhibition of the release of **(B)** IL-5, **(C)** IL-8, and **(D)** TNF-α induced by anti-IgE. IgE-sensitized human cultured mast cells were pre-incubated with 10 µg/ml cRAD or cRGD for 15 min at room temperature and then aliqouted onto 1 µg/ml of immobilized hOPN for 1 h incubation, which was followed by 1 µg/ml anti-IgE stimulation for 2 or 24 h for adhesion and cytokines release, respectively. The percentage of cell adhesion was then determined as described in Figure 2. The results of cytokine release are expressed as the percentage of the anti-IgE induced level of cytokine released that was reduced in the presence of immobilized osteopontin [inhibition (%)]. Data are shown as mean ± SEM (*n* = 3); *p < 0.05, **p < 0.01 by paired *t*-test when compared with values obtained with phosphate-buffered saline.

Besides monoclonal antibodies, the cyclic pentapeptide cyclo(-Arg-Gly-Asp-D-Phe-Val-) (cRGD), a highly potent and selective inhibitor for integrin αvβ3 also demonstrated significant suppression of the mast cell modulating actions of immobilized hOPN but not the control peptide cRAD (Figure 5). Following incubation with 10 µg/ml of cRGD, the degree of HCMC adhesion was reduced from 31.3 ± 0.9 to 21.7 ± 0.7% while the inhibition of anti-IgE induced release of IL-5, IL-8, and TNF-α were respectively reduced from 18.3 ± 1.5, 16.7 ± 0.8% and 40.9 ± 4.9 to 11.0 ± 1.7%, 5.7 ± 1.3 and 16.6 ± 3.3%.

## Discussion

We have demonstrated for the first time that OPN can modulate human mast cell functions, but only in the immobilized adhesion molecule configuration, not when it is in the soluble cytokine configuration. The immobilized OPN facilitated the adhesion of anti-IgE-activated HCMC while suppressing the subsequent syntheses of cytokines, including IL-5, IL-8, and TNF-α but with no effect on the immediate release of preformed granular mediators such as histamine. Our results are contradictory to those reported by Nagasaka et al. who employed mast cells cultured from fetal skin of OPN knockdown mouse and soluble mouse recombinant OPN (3). They discovered that OPN in solution could induce chemotaxis and enhance anti-IgE-induced degranulation of murine mast cells. The discrepancy between the two studies provides further evidence illustrating the well-recognized concept of mast cell heterogeneity and the importance of employing mast cells of human origin for clinically relevant results (13). Furthermore, our results suggested that future studies investigating the contribution of OPN to the pathogenesis of inflammation should include immobilized OPN in addition to the commonly employed soluble OPN.

The level of OPN is increased during inflammation through *de novo* synthesis and release by resident epithelial, endothelial, and smooth muscle cells as well as a host of infiltrating immune cells (10). Both pro- and anti-inflammatory actions have been reported for OPN suggesting that the isoforms of OPN, the immune cells, and the inflammatory reactions studied all contribute to the overall final outcome (14). In addition to existing in soluble or matrix-affixed form, OPN undergoes substantial post-translational modifications with different degrees of phosphorylation and glycosylation (15, 16). Our preliminary studies had compared the effect of recombinant hOPN and milk hOPN on human mast cell adhesion and found that only the milk-derived protein demonstrated positive results, suggesting the importance of *in vivo* post-translation modification in determining the functions of OPN. The failure of soluble hOPN to induce significant actions on HCMC implicates that the RGD region of the protein is not normally exposed for the interaction with integrin receptors on HCMC surface. Our results suggested that attachment to the culture well surface orientated the hOPN molecule in such a way that the integrin interacting region is exposed to enhance HCMC adhesion and the subsequent functional modulation. In fact, the orientation of OPN following attachment to biomaterial surfaces has been demonstrated to be an important factor in determining the biological functions of OPN (17). Furthermore, the activation of HCMC by anti-IgE seems to facilitate the conformation change of the OPN receptors on the cell surface to enhance their interaction with OPN as demonstrated by the enhanced adhesion and the cellular shape change when HCMC were activated in the presence of immobilized OPN. Similar results had been reported for the adhesion of platelets to OPN-coated beads (18). It was found that activation of platelets by ADP could increase the force of interaction between individual platelet αVβ3 and OPN molecules, suggesting that the mechanism responsible for platelet adhesion to OPN-coated surfaces required an agonist-induced increase in the affinity of αVβ3 for OPN through the alteration of the receptor conformation in consequence to intracellular signaling initiated during platelet activation.

Immunohistochemical studies revealed that HCMC expressed integrin αvβ3, α5, β2, β4, and CD44. Further studies with neutralizing antibodies of these receptors revealed that only the antibody against integrin αvβ3 could reverse both the enhanced adhesion and the inhibition of cytokine release of anti-IgE-activated HCMC mediated by immobilized hOPN, suggesting that integrin αvβ3 is the main functional cell surface receptor that interacts with hOPN. However, α5 and β2 may also contribute to the regulatory action of OPN on HCMC cytokine release as the inhibitory effect of hOPN on anti-IgE induced release of cytokines was also reversed by the antibodies against integrin α5 and β2. The inconsistency in the reversing effect of integrin antibodies on immobilized hOPN-mediated adhesion and cytokine release suggests that the suppressing effect of immobilized hOPN on anti-IgE-activated HCMC is not necessarily dependent on adhesion. The failure of the antibody against CD44 to reverse any of the mast cell modulatory actions of immobilized hOPN despite of the positive immunostaining of HCMC for CD44 suggested that the CD44 isoform expressed is not the variants that optimally interact with OPN. Human mast cells have been demonstrated to express only the standard form of CD44 under normal conditions (19), while the CD44 variant 6 and 7 have been identified to interact with OPN in inflammation and cancer (20). The significant contribution of integrin to the mast cell modulating action of OPN is further confirmed by cyclic RGD which has been shown to block the interaction between OPN and αvβ3, αvβ5 and α5β1 (21). This peptide effectively reversed the enhanced adhesion and suppressed cytokine release in anti-IgE-activated HCMC mediated by immobilized hOPN, which is consistent with the results of neutralization of integrin αvβ3 and α5 by their respective monoclonal antibodies.

In summary, our studies have demonstrated that OPN does not suppress human mast cell degranulation which contributes to the acute phase of allergic reaction but is capable of suppressing IgE-mediated release of cytokines from human mast cells that maintains the chronic phase of allergic reactions. While the results are contradictory to those observed in mouse mast cells (3), they are in accordance to *in vivo* studies employing OPN knockout mice and OPN antibodies which suggested that OPN could suppress allergic reactions (22, 23). The exacerbation of allergic airway diseases following neutralization of OPN at the time of allergen challenge suggests that the increased expression of OPN in allergic diseases might be an inherent protective mechanism (24). In addition to the previously suggested suppression of Th2 cells through increased plasmacytoid dendritic cells proliferation (24), direct inhibition of mast cell pro-inflammatory cytokine release by immobilized OPN as reported here may be part of this mechanism. Another important physiological function of OPN is the regulation of tissue fibrosis and wound healing (4, 25). Mast cells are also known to contribute to wound healing through the release of serine proteases and angiogenesis factors (26, 27), and thus the adhesion of mast cells to immobilized OPN facilitates the accumulation of mast cells at tissue injury sites where expression of OPN is increased. The failure of hOPN to reduce basal and anti-IgE induced release of VEGF from HCMC as observed in this study suggests that the modulatory action of immobilized OPN may suppress the initially pro-inflammatory functions of human mast cells at injury sites and allow other functions of these cells such as tissue repairing to become more prominent at the later stage of inflammation. Taking together, our studies have provided evidence for a new direction to studying in humans the interaction between OPN anchored in the extracellular matrix of inflamed tissues and tissue mast cells in the context of tissue repair and wound healing. Further investigations such as how immobilized OPN modulates the expression of other tissue repairing factors, such as metalloproteinase in human mast cells are certainly required to better understand the pathophysiological significance of the interaction between these two abundant elements at tissue injury sites which underlay the pathogenesis of not only chronic inflammatory diseases but also various forms of tumor.

## Author Contributions

CN: planning and performing experiments, data collection, data analysis, making figures, and writing manuscript. IT: data analysis and reviewing manuscript. SS: mast cell culturing and morphology study. YY: data analysis. HL: planning the study and supervision of writing manuscript.

## Conflict of Interest Statement

None of the authors have any conflict of interest, nor have they received any money for this study. Research is part of their daily activities. All authors had full access to all data and take responsibility for the integrity and accuracy of the data analysis.
